# Multiple human pressures and their spatial patterns in European running waters

**DOI:** 10.1111/j.1747-6593.2011.00285.x

**Published:** 2011-09-19

**Authors:** Rafaela Schinegger, Clemens Trautwein, Andreas Melcher, Stefan Schmutz

**Affiliations:** Institute of Hydrobiology and Aquatic Ecosystem Management, Department of Water, Atmosphere and Environment, BOKU – University of Natural Resources and Life SciencesVienna, Austria

**Keywords:** impact assessment, river, water framework directive, water quality

## Abstract

Running water ecosystems of Europe are affected by various human pressures. However, little is known about the prevalence, spatial patterns, interactions with natural environment and co-occurrence of pressures. This study represents the first high-resolution data analysis of human pressures at the European scale, where important pressure criteria for 9330 sampling sites in 14 European countries were analysed. We identified 15 criteria describing major anthropogenic degradation and combined these into a global pressure index by taking additive effects of multiple pressures into account. Rivers are affected by alterations of water quality (59%), hydrology (41%) and morphology (38%). Connectivity is disrupted at the catchment level in 85% and 35% at the river segment level. Approximately 31% of all sites are affected by one, 29% by two, 28% by three and 12% by four pressure groups; only 21% are unaffected. In total, 47% of the sites are multi-impacted. Approximately 90% of lowland rivers are impacted by a combination of all four pressure groups.

## Introduction

Recent studies in Europe and elsewhere emphasise that numerous human alterations and impacts (herein referred to as pressures) directly affect the physico-chemical conditions of running waters and strongly influence aquatic biota. According to Tockner *et al*. (2009), nearly all European river basins are heavily affected by human activities, that is, the degradation of European rivers and streams is widespread. A key pressure is water pollution (FAME Consortium 2004; Degerman *et al*. 2007). Hydrological alterations such as impoundment (Reid 2004), water abstraction (Pyrce 2004) and hydropeaking (Flodmark *et al*. 2004) are also known to degrade aquatic biota. Morphological alterations such as channelisation and riverbed degradation cause severe impacts such as habitat degradation and loss (Raat 2001; Aarts *et al*. 2004). Dams are generally known for their impacts at the catchment scale, with both upstream and downstream effects stemming from inundation, flow manipulation and fragmentation (Nilsson *et al*. 2005). Furthermore, the disruption of both longitudinal and lateral connectivity significantly impairs aquatic biota, particularly fish (Rieman & Dunham 2000; Hughes & Rood 2003). Finally, these pressures also impact the assimilative capacity of running waters, that is, when the assimilative capacity is reduced, the impact of further pressures can be even greater (Roux *et al*. 1999).

Because of the traditional focus on studies at the local or national level, we lack a common understanding of pressures on a large spatial extent, for example, across Europe. However, the European Water Framework Directive (WFD, European Commission 2000) requires a consistent and comparable ‘identification of significant anthropogenic pressures and the assessment of their impacts on water bodies’ (ANNEX II WFD, European Commission 2000).

According to Vinebrooke and Cottingham (2004), pressures often have additive and multiplicative effects. An Institute for Environment and Sustainability (IES) report (Solimini *et al*. 2006) indicates that multiple pressures act simultaneously in most cases, requiring managers to define a hierarchy amongst these to identify priority actions. Moreover, a better understanding of the distinct effects of single pressures, multiple pressures and their interactions is a clear precondition for effective river restoration (Palmer *et al*. 2005; Wohl *et al*. 2005; Schmutz *et al*. 2007) as well as effective catchment management. Finally, pressures are predicted to intensify in the future because of an increase in extreme flow events and the growing water demand for agriculture and energy (European Commission 2009). However, only a few studies have examined the relationships between various pressures, and our knowledge of the co-occurrence and the interactions of pressures, particularly at larger scales such as Europe, is poor.

The objectives of this paper are to analyse various pressure types across Europe based on a large and unique data set to achieve the following: (1) identify important pressure groups; (2) detect dominating pressures; (3) elucidate prevailing pressure combinations (chemical–physical pressures versus hydromorphological pressures); and (4) detect spatial patterns across Europe [ecoregions versus river types (RTs)].

## Methods

### Data set and pressure information

Out of a large database (EFI+ Consortium 2009), we selected a data set with 9330 sites (Fig. 1) on approximately 3100 rivers within 14 European countries and 10 ecoregions (Illies, 1978; Table 1). We did not consider the ecoregions ‘Baltic province’, ‘Borealic uplands’, ‘Pontic province’, ‘The Pyrenees’, ‘Italy and Corsica’ or ‘The Carpathians’ for further analyses because of the low number of sites (< 100 for each ecoregion) and the patchy spatial distribution of the data. The environmental characteristics for each ecoregion encompass mean annual air temperature, Strahler order (a measure used to define river size based on a hierarchy of tributaries, Strahler 1952), slope, altitude, upstream size of catchment and distance from the source (Table 2). The data sources and methods have been published by the EFI+ Consortium (2007).

**Figure:**
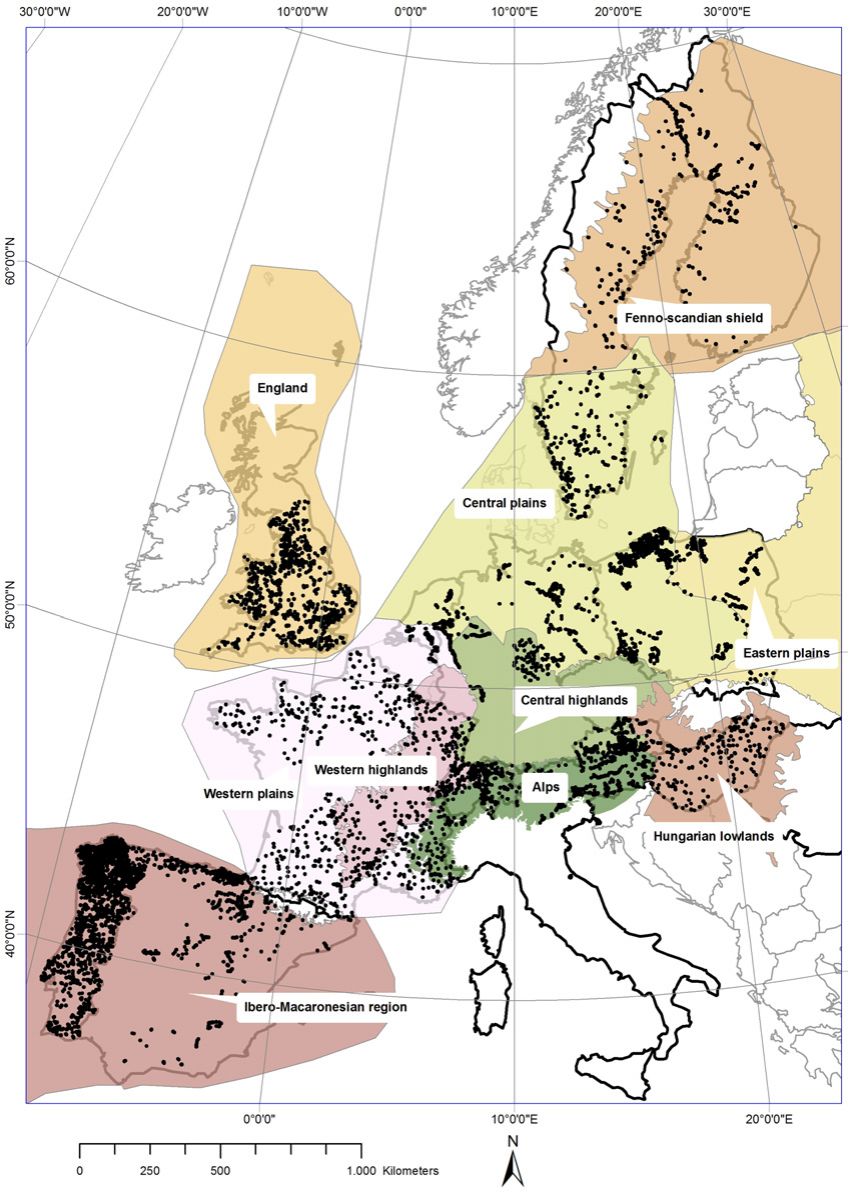
Location of the 9330 sampling sites spread over 10 ecoregions in Europe.

**1:** Number of analysed sites per country and ecoregion

Ecoregion (number of ecoregion)	Country															
	AT	CH	DE	ES	FI	FR	HU	IT	NL	PL	PT	RO	SE	UK	Total
Alps (4)	373	207	0	0	0	52	0	91	0	0	0	0	0	0	723
Central highlands (9)	437	2	289	0	0	21	0	0	0	24	0	0	0	0	773
Central plains (14)	0	0	470	0	0	0	0	0	110	590	0	0	332	0	1502
Eastern plains (16)	0	0	0	0	0	0	0	0	0	227	0	85	0	0	312
England (18)	0	0	0	0	0	0	0	0	0	0	0	0	0	1228	1228
Fenno-scandian shield (22)	0	0	0	0	266	0	0	0	0	0	0	0	211	0	477
Hungarian lowlands (11)	63	0	0	0	0	0	191	0	0	0	0	0	0	0	254
Ibero-Macaronesian region (1)	0	0	0	2075	0	1	0	0	0	0	923	0	0	0	2999
Western highlands (8)	0	280	0	0	0	241	0	0	0	0	0	0	0	0	521
Western plains (13)	0	0	22	1	0	446	0	0	72	0	0	0	0	0	541
Total	873	489	781	2076	266	761	191	91	182	841	923	85	543	1228	9330

**2:** Standard deviation (SD), median and range of environmental variables in ecoregions

Illies ecoregion (number of ecoregion)		Air temperature (°C)	Strahler order (1–12)	River slope (‰)	Altitude (m.a.s.l.)	Size of catchment (km^2^)^a^	Distance from river source (m)
Alps (4)	SD	1.8	1.4	52.0	373.4	1 254.1	49.7
Median	7.6	4.0	10.5	621.0	92.0	15.0
Range	0.2–12.1	1–7	0.06–387	0.01–2111	0–10 017	0–435
Central highlands (9)	SD	1.0	1.9	7.3	185.2	27 288.0	219.0
Median	8.6	4.0	2.9	301.0	351.0	41.0
Range	5.3–10.4	1–8	0.1–113	25–856	1–147 809	0–984
Central plains (14)	SD	1.1	2.2	5.9	71.0	42 204.3	277.4
Median	8.0	3.0	1.0	44.0	209.5	27.0
Range	4.4–10.2	1–8	0.01–76.6	0–370	2–10 469.3	0–1080
Eastern plains (16)	SD	0.8	1.7	3.4	91.3	36 123.5	174.6
Median	7.6	3.0	1.0	107.0	513.0	53.5
Range	6.4–10.2	1–8	0.01–27.9	0–452	4–195 208	2–988
England (18)	SD	0.7	1.1	17.8	74.8	527.0	27.3
Median	9.6	2.0	3.1	49.0	70.0	16.0
Range	6.8–10.8	1–6	0.01–242.9	0–353	1–9653	1–252
Fenno-scandian shield (22)	SD	1.7	1.6	9.4	109.9	8196.5	116.7
Median	1.3	3.0	2.7	129.0	693.0	68.0
Range	−2.3–5.2	1–6	0.1–79.3	1–480	2–40 157	2–530
Hungarian lowlands (11)	SD	0.7	1.8	4.0	70.5	6 916.5	409.2
Median	10.0	3.5	1.3	128.0	100.0	39.0
Range	8–11.3	1–8	0.1–32.7	79–387	0–100 161	0–1454
Ibero-Macaronesian region (1)	SD	2.0	1.4	23.7	315.0	7 187.2	88.7
Median	13.4	3.0	8.5	320.0	77.0	18.0
Range	6.9–18.2	1–8	0.01–774	1–1485	1–96 303	1–981
Western highlands (8)	SD	1.2	1.3	18.4	211.0	4 406.6	91.3
Median	9.3	2.0	7.9	441.0	45.0	11.0
Range	6.1–13.6	1–8	0.036–165.4	140–1340	0–51 500	0–1380
Western plains (13)	SD	1.3	1.9	9.3	167.4	40 250.5	247.9
Median	10.7	3.0	2.0	84.0	214.0	29.0
Range	8.3–15.6	1–8	0.01–63	0–865	2–185 000	1–1078

a River catchment located between the sampling site and the mouth of river into the sea.

Our data set contained 15 pressure variables assigned to four groups, that is, hydrology, morphology, water quality and connectivity. The pressure variables (names given in brackets) were selected according to known effects on aquatic habitats and organisms (Table 3): In impounded rivers (H_imp), loss of fluvial habitat, embeddedness of substrate and altered channel form (Reid 2004). In sites affected by hydropeaking (H_hydrop), ramping rates and discharge changes result in the mortality of fish and benthic invertrebrates because of stranding and desiccation (Flodmark *et al*. 2004; Scruton *et al*. 2008). Sites affected by water abstraction (H_waterabstr) show a decrease in channel maintenance flow as well as geomorphic and water quality impacts (Reid 2004; Thorstad *et al*. 2008; Benejam *et al*. 2009). The flushing of reservoirs (H_resflush) results in increased concentrations of suspended sediment, which impacts fish and invertebrate fauna (Bash & Berman 2001; Crosa *et al*. 2010). In addition, toxic effects may occur, as many substances (e.g. heavy metals) can be attached to the sediments; these substances can be released with the sediments further causing water quality problems. A seasonal hydrograph modification (H_hydromod) may occur because of hydrological alteration caused by water storage for irrigation or hydropower resulting in changes of channel morphology and physical habitat composition (Jackson & Marmulla 2000, Roy *et al*. 2005). Channelisation (M_channel) is the alteration of natural morphological channels into straightened rivers. This alteration reduces habitat heterogeneity and causes riverbed degradation (Raat 2001; Aarts *et al*. 2004; Muhar *et al*. 2008). A change of natural channel cross-sections (M_crosssec) into technical cross-sections or U-profiles results in habitat degradation (Bravard *et al*. 1997; Raat 2001; Muhar *et al*. 2008). Finally, altered instream habitat (M_instrhab), that is, lack of woody debris, gravel bars and pools, also affects habitat quality (Bain & Stevenson 1999; Muhar *et al*. 2008). As the latter three variables all describe morphological instream habitat changes, they were aggregated into M_morhph_instr by calculating the arithmetic mean of the original variables M_channel, M_instrhab and M_crossec as follows:

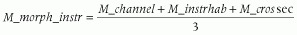


**3:** Definition and classification of pressure information within four groups: hydrology (H), morphology (M), water quality (W) and connectivity (C)

Pressure variable	Group	Code	Explanation; short description of classes	Examples of effects
Impoundment	HPI	H_imp	Natural flow velocity reduction on site because of impoundment; 1 = no (no impoundment), 3 = weak, 5 = strong	Reid (2004)
Hydropeaking	HPI	H_hydrop	Site affected by hydropeaking; 1 = no (no hydropeaking), 3 = partial, 3 = yes	Flodmark *et al*. (2004); Scruton *et al*. (2008)
Water abstraction	HPI	H_waterabstr	Site affected by water flow alteration/minimum flow; 1 = no (no water abstraction), 3 = weak to medium (less than half of the mean annual flow), 5 = strong (more than half of mean annual flow)	Reid (2004)); Thorstad *et al*. (2008); Benejam *et al*. (2009)
Reservoir flushing	HPI	H_resflush	Fish fauna affected by flushing of reservoirs upstream of site; 1 = no, 3 = yes	Bash & Berman (2001); Crosa *et al*. (2010)
Hydrograph modification	HPI	H_hydromod	Seasonal hydrograph modification because of hydrological alteration (water storage for irrigation, hydropower etc.); 1 = no, 3 = yes	Jackson & Marmulla (2000); Roy *et al*. (2005)
Channelisation^a^	MPI	M_channel	Alteration of natural morphological channel plan form; 1 = no, 3 = intermediate, 5 = straightened	Raat (2001); Aarts *et al*. (2004); Muhar *et al*. (2008)
Cross-section^a^	MPI	M_crosssec	Alteration of cross-section; 1 = no, 3 = intermediate, 5 = technical crossec./U-profile	Bravard *et al*. (1997); Raat (2001); Muhar *et al*. (2008)
Instream habitat^a^	MPI	M_instrhab	Alteration of instream habitat conditions; 1 = no, 3 = intermediate, 5 = high	Muhar *et al*. (2008)
Embankment	MPI	M_embankm	Artificial embankment; 1 = no (natural status), 2 = slight (local presence of artificial material for embankment), 3 = intermediate (continuous embankment but permeable), 5 = high (continuous, no permeability)	Neumann (2002), Wolter (2008)
Flood protection	MPI	M_floodpr	Presence of dykes for flood protection; 1 = no, 3 = yes	Tockner *et al*. (1998); deLeeuw *et al*. (2007)
Barriers segment upstream	CPI	C_B_s_up	Barriers on segment level upstream; 1 = no, 3 = partial, 3 = yes	Rieman & Dunham (2000); Zitek *et al*. (2008)
Barriers segment downstream	CPI	C_B_s_do	Barriers on segment level downstream; 1 = no, 4 = partial, 4 = yes	Rieman & Dunham (2000); Zitek *et al*. (2008)
Acidification	WQPI	W_acid	Acifidication; 1 = no, 3 = yes	Sandin & Johnson (2000); Leduc *et al*. (2008)
Eutrophication	WQPI	W_eutroph	Artificial eutrophication; 1 = no, 3 = low, 4 = intermediate (occurrence of green algae), 5 = extreme (oxygen depletion)	Sandin & Johnson (2000); Smith *et al*. (2006)
Organic pollution	WQPI	W_opoll	Is organic pollution observed; 1 = no, 3 = weak, 5 = strong	Penczak & Kruk (1999); Nilsson & Malm Renöfält (2008)

a Original variables M_channel, M_instrhab and M_crossec aggregated to M_morph_instr.

Artificial embankment (M_embank), from local presence to continuous embankment with no permeability, is thought to impact lateral connectivity and the quality of shoreline and riverbank habitats (Neumann 2002; Wolter 2008). The presence of dykes for flood protection (M_floodpr) affects riparian habitat and floodplain hydrology and habitat (Tockner *et al*. 1998; deLeeuw *et al*. 2007). Barriers on the segment up/downstream (C_B_s_up, C_B_s_do) and on the catchment level influence the degree of habitat fragmentation and migration pathways (Rieman & Dunham 2000; Zitek *et al*. 2008). Related to water quality pressures, acidification (W_acid) in a river (caused by air pollution and resulting acid rain) leads to low pH values (Sandin & Johnson 2000; Leduc *et al*. 2008), causing increased physiological stress and reducing the survival of fish. Increased N and *P* rates result in artificial eutrophication (W_eutroph), causing algal blooms and oxygen depletion (Sandin & Johnson 2000; Smith *et al*. 2006). Finally, organic pollution (W_opoll) influences dissolved oxygen concentration, biological oxygen demand and chemical oxygen demand (Penczak & Kruk 1999; Nilsson & Malm Renöfält 2008).

Pressure data were collected based on national databases compiled for the Water Framework Directive (WFD, Article 5 'Characteristics of the river basin district'); regional and national monitoring; detailed protocol data from field mappings; information from national and regional water authorities; and expert judgement (Schinegger et al. 2008; EFI+ Consortium 2009). Depending on the type of pressure and sources available, the information was provided in different formats, that is, binary as presence/absence information or as ordinal data ranging from three to five modalities (Table 3). To overcome the inequity in the number of modalities, we defined an ordinal ranking scheme and harmonised all pressure parameters along a gradient ranging from 1 (nearly undisturbed) to 5 (strongly impacted, Table 3).

Information on connectivity pressures was collected on the segment scale: 1 km for small rivers (catchment < 100 km^2^), 5 km for medium-sized rivers (catchment 100–1000 km^2^) and 10 km for large rivers (catchment > 1000 km^2^). A segment for a small river was thus 500 m upstream and 500 m downstream of the sampling site. Connectivity pressure on the catchment level was defined as any migratory barrier located between the sampling site and the mouth of a river into the sea. However, as most of the sites (85%) are disconnected from the sea, this type of pressure was not considered for further analyses.

### Dominating pressure groups, global pressure index (GPI) and multiple pressures

To evaluate the pressure status of European rivers in terms of individual pressures, we calculated four pressure type specific indices, that is, one each for hydrology (HPI), morphology (MPI), water quality (WQPI) and connectivity (CPI). These indices were calculated by averaging the single pressure parameter values of classes 3, 4 and 5 to avoid that values < 3 compensate for values ≥ 3: 
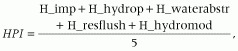


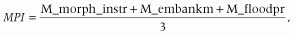


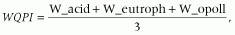


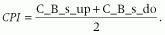


For example, a site without acidification (W_acid = 1), with low eutrophication (W_eutroph = 3) and with strong organic pollution (W_opoll = 5) would receive a value of 4 for the WQPI instead of 3 when simply using the mean of all values. In a second step, we calculated the number of pressure groups affected (‘affected_groups’). This value varied from 1 to 4 depending on how many of the four pressure type specific indices HPI, MPI, WQPI and CPI were higher than or equal to 3.0. Afterwards, to express the degradation of a site by multiple pressures in one single index value, we calculated a GPI as follows: 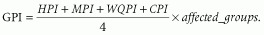


The GPI varied from 0 to 20 and was rescaled in four classes according to the number of pressure groups involved: class 0 – unimpacted/slightly impacted sites; class 1 – values ranging from 3 to 5 (single pressure sites); class 2 – values ranging from 6 to 8 (double pressure sites); class 3 – values ranging from 9 to 11 (triple pressure sites) and class 4 – values ranging from 12 to 20 (quadruple pressure sites).We are aware that the calculation of such an index goes along with a reduction of dimensions and information; however, the GPI can be a helpful tool to illustrate the cumulative effects of pressures.

In addition, to determine the proportion of sites affected by hydromorphological pressures versus a combination with water quality pressures, we split our pressure data into three groups: only water quality pressures (W); hydromorphological pressures including connectivity (HMC); and a combination of both (W + HMC).

Finally, to check the relationship between (multiple) pressures and human population density, the density of inhabitants/km^2^ (European Environment Agency 2005) in a buffer of 10 km around each sampling site was calculated using GIS software (ArcGIS Desktop 9.3, ESRI © 2008).

### RTs

To analyse the spatial patterns of pressures related to environmental and ecological characteristics, we used the European fish types (Melcher *et al*. 2007). These were calculated using Excel-based software developed by the FAME Consortium (2004), which is available at http://fame.boku.ac.at/. This model links key environmental characteristics of European running waters (i.e. altitude, distance from the source, wetted width, mean annual air temperature, slope, latitude and longitude) to fish assemblages to predict assemblages typical for unimpacted sites. For our purposes, a simplified typology was derived by aggregating the original 15 EFT into six RTs. Types 1 to 4 represent rivers dominated by *Salmo trutta fario*, varying in the amount of the accompanying species (RT A-Headwater). Types 5 and 6 represent downstream river sections with lower gradients dominated by *Phoxinus phoxinus* (RT B-Downstream). *Thymallus thymallus* is mainly found in Types 7 and 9 (RT C-Greyling). Types 8, 11 and 12 are dominated by anadromous and potamodromous salmonids, that is, *Salmo salar*, *Salmo trutta lacustris* and *Salmo trutta trutta* (RT D-Salmon). Types 10 and 13 represent southern fish assemblages, with the latter characterised by Mediterranean endemics (RT E-Mediterranean). Types 14 and 15 are lowland rivers dominated by *Gasterosteus aculeatus* and *Rutilus rutilus* (RT F-Lowland). The relationship between ecoregions and RTs is shown in Table 4.

**Table 4:** Distribution of sites (N = 9330) within ecoregions and river types

Ecoregion (Nr.)	A-Headwater	B-Downstream	C-Greyling	D-Salmon	E-Mediterranean	F-Lowland	Total
Ibero-Macaronesian region (1)	46.0%	41.9%			11.3%	0.8%	100.0%
Alps (4)	67.8%	3.4%	24.3%	0.4%		4.1%	100.0%
Western highlands (8)	77.8%	11.5%				10.7%	100.0%
Central highlands (9)	34.2%	11.7%	15.7%			38.4%	100.0%
Hungarian lowlands (11)	16.4%	72.5%	5.3%			5.8%	100.0%
Western plains (13)	31.6%	36.7%		0.4%	4.3%	27.1%	100.0%
Central plains (14)	15.0%	31.5%		25.6%		27.9%	100.0%
Eastern plains (16)	6.6%	85.1%		1.0%		7.3%	100.0%
England (18)	11.2%	83.0%		5.8%		0.1%	100.0%
Fenno-scandian shield (22)	53.0%	5.1%	1.1%	40.9%			100.0%
Total	36.5%	38.5%	3.4%	7.0%	4.0%	10.7%	100.0%

## Results

### Dominating pressure groups

The results for pressure type specific indices showed that water quality pressures were present in 59% of the sites (WQPI classes 3–5, Fig. 2a), with the worst conditions for sites in the following ecoregions (ecoregion numbering according to Table 1): Central highlands (9), Hungarian lowlands (11), Western highlands (8) and Western plains (13). In terms of hydrological pressures, impacts were observed for 41% of the sites (Fig. 2b), with the worst conditions in the Central highlands (9) and Western plains (13). Morphological habitat degradation was frequent. Impaired conditions were found for 39% of the sites (Fig. 2c), with the worst conditions in the Alps (4), the Central highlands (9) and the Hungarian lowlands. Connectivity pressures on the segment scale were determined for 35% of the sites (Fig. 2d), with the worst conditions in the Alps (4), the Central highlands (9) and the Western highlands (8). On the catchment scale (the river catchment from sampling site to mouth into sea), more than 85% of the sites were affected by connectivity pressures.

**Figure:**
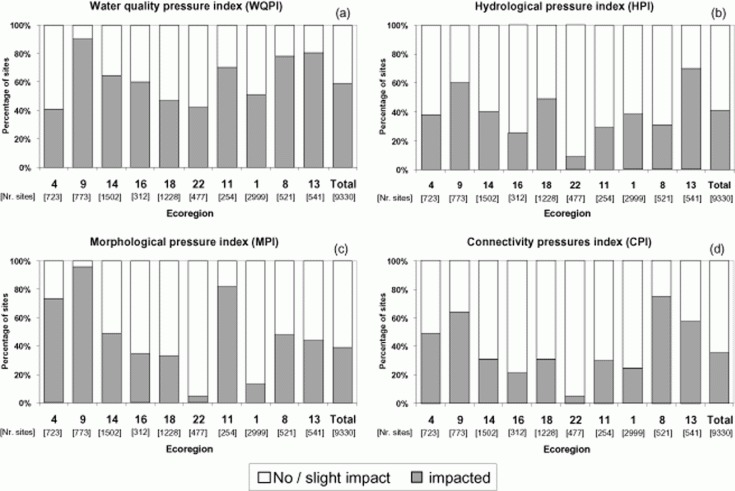
Percentage of impacted sites per ecoregion: water quality pressures (a), hydrological pressures (b), morphological pressures (c) and connectivity pressures (d). Black bars represent impacted sites (index classes 3, 4 and 5) and white bars unimpacted/slightly impacted sites (class < 3). 1 = Ibero-Macaronesian region, 4 = Alps, 8 = Western highlands, 9 = Central highlands, 11 = Hungarian lowlands, 13 = Western plains, 14 = Central plains, 16 = Eastern plains, 18 = England, 22 = Fenno-scandian shield.

### Multiple pressures in ecoregions and RTs

As Fig. 3 illustrates, the degradation of European rivers is widespread, as more than 79% of the sites were impacted. Approximately 12% were affected only by water quality problems (W), but 47% were also associated with other pressures. Approximately 20% were affected only by hydromorphological pressures (HMC), and 21% were slightly/not affected (NoP). Multiple pressure analysis also demonstrated that the patterns and relationships vary throughout Europe's ecoregions (Fig. 3). Hydromorphological pressures without significant water quality pressures (HMC) were frequent in approximately 50% of the Alpine sites (4). In contrast, water quality pressures (W) were found at approximately 40% of the Fenno-scandian (22) sites, mainly because of acidification. Hydromorphological pressures combined with water quality pressures (W + HMC) were most frequent for sites in the Central highlands (9), Central plains (14), Hungarian lowlands (11), Western highlands (8) and Western plains (13); more than 50% of the sites were affected within the latter.

**Figure:**
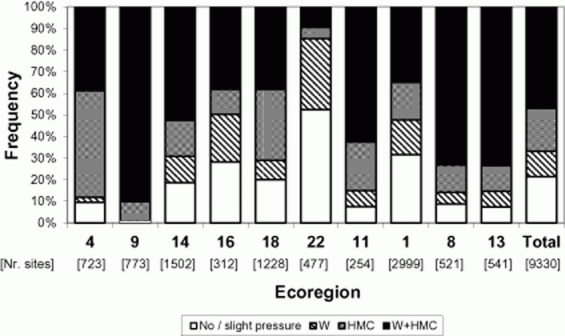
Proportion of sites unimpacted/slightly impacted (no/slight pressure), affected by water quality pressures only (W), by hydromorphological pressures only (HMC) and by a combination of water quality and hydromorphological pressures (W + HMC) per ecoregion and the total. 1 = Ibero-Macaronesian region, 4 = Alps, 8 = Western highlands, 9 = Central highlands, 11 = Hungarian lowlands, 13 = Western plains, 14 = Central plains, 16 = Eastern plains, 18 = England, 22 = Fenno-scandian shield.

Analyses of pressures within the RTs (Fig. 4) showed that sites in RT A – Headwaters and RT E – Mediterranean were associated with the category unimpacted/slightly impacted (approximately 36% and 30% of the sites, respectively). In RT C-Greyling, the sites were exclusively affected by hydromorphological pressures (39% of sites) and by a combination with water quality pressures (55%). RT D-Salmon was affected by a combination of all pressures (38% of sites) as well as water quality pressures exclusively (28%). In RT F-Lowlands, 90% of the sites were affected by a combination of all pressures, and only 4% were without or had only slight pressures. Headwaters were generally less impacted than lowland rivers, and they provided more reference sites and fewer multi-impacted sites. The results also show that headwaters are often affected by hydromorphological pressures and that water quality pressures were less important in this RT than in lowland rivers.

**Figure:**
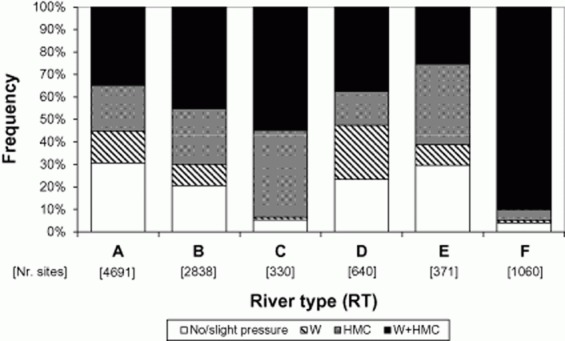
Proportion of sites affected by different pressure combinations in river types: A = Headwater, B = Downstream, C = Greyling, D = Salmon, E = Mediterranean, F = Lowland. Unimpacted/slightly impacted (no/slight pressure), water quality pressures only (W), hydromorphological pressures only (HMC) and a combination of water quality and hydromorphological pressures (W + HMC).

### GPI

Figure 5 illustrates the GPI in classes 0 to 4 from no/slight pressure to quadruple pressure.

**Figure:**
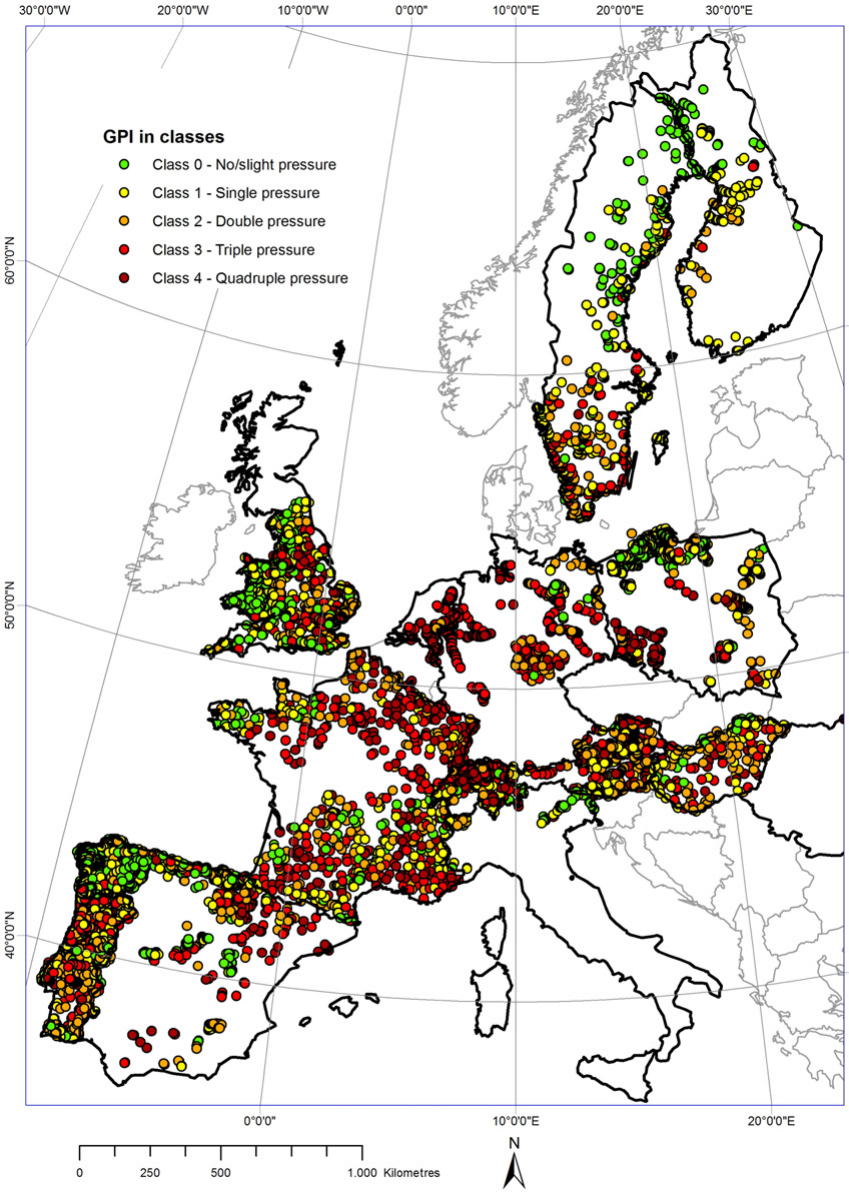
Distribution of the global pressure index (GPI) for sampling sites.

In Tables 5 and 6, the values of the GPI in ecoregions and RTs are shown.

**Table 5:** Distribution of the global pressure index (GPI) in ecoregions

Ecoregion (Nr.)	GPI			
Min	Max	Mean	Median
Ibero-Macaronesian region (1)	3.0	15.5	6.2	6.0
Alps (4)	3.0	18.5	8.0	7.3
Western highlands (8)	3.0	18.5	9.4	9.9
Central highlands (9)	3.0	16.5	10.6	10.3
Hungarian lowlands (11)	3.0	15.5	7.9	7.6
Western plains (13)	3.0	17.0	9.4	9.5
Central plains (14)	3.0	17.0	7.8	7.3
Eastern plains (16)	3.0	16.6	6.8	6.0
England (18)	3.0	18.0	7.5	7.5
Fenno-scandian shield (22)	3.0	10.0	4.0	3.0

**Table 6:** Distribution of the global pressure index (GPI) in river types

River type	GPI			
Min	Max	Mean	Median
A-Headwater	3.0	18.5	6.8	6.0
B-Downstream	3.0	18.0	7.4	7.0
C-Greyling	3.0	15.7	9.8	9.7
D-Salmon	3.0	16.5	6.1	6.0
E-Mediterranean	3.0	15.2	5.8	6.0
F-Lowland	3.0	17.0	10.9	10.3

Figure 6 shows the relationship between population density and pressure types. Compared with the category no/slight pressure, the combined pressures (water quality pressures in combination with hydromorphological pressures) tend to be found in areas with higher population density. However, there are also sites that have low population density, but high hydromorphological or combined pressures.

**Figure:**
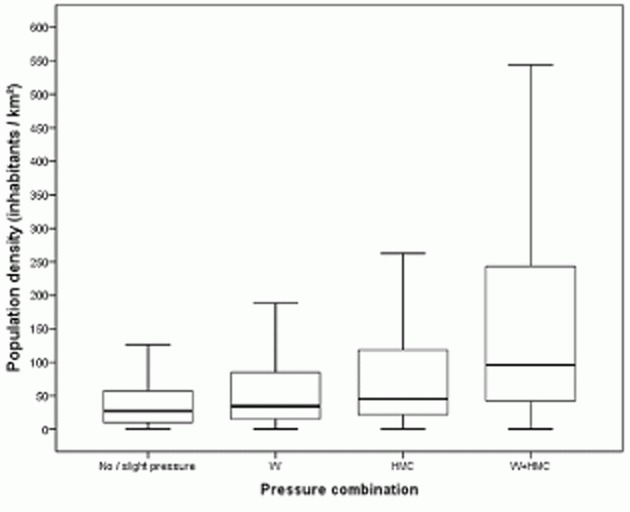
Relationship between population density and pressure combination. Unimpacted/slightly impacted (no/slight pressure), water quality pressures only (W), hydromorphological pressures only (HMC) and a combination of water quality and hydromorphological pressures (W + HMC).

## Discussion

This study represents the first high-resolution data analysis of human pressures on running waters at the European scale. Degerman *et al*. (2007) already worked on a pan-European classification of environmental degradation caused by chemical, physical and biological pressures in a similar study (FAME Consortium 2004). However, their work was mainly based on expert judgement; only seven criteria were available to generate a single pressure variable, and their data set contained fewer sites (approximately 7000). In contrast, our intent was to describe the impacts of hydrological, morphological, water quality and connectivity pressures by calculating pressure type specific indices. Further, we identified combined pressures and compared them with different ecoregions and RTs. Finally, we calculated a GPI to quantify the overall pressure status of a specific site by a single index value, including the additive effects of multiple pressures.

### Dominating pressure groups and multiple pressures

In terms of connectivity pressures, Tockner *et al*. (2009) reported that natural flow in European rivers is disrupted by over 6000 major dams, and Degerman *et al*. (2007) detected connectivity disruptions for 60% of the sites they analysed. In our study, 35% of all sites were affected by connectivity pressures (CPI) on the river segment scale, and 85% of the sites were affected on the catchment scale. In terms of hydrological (HPI) and morphological pressures (MPI), we found impacts for 41 and 39% of the sites, respectively. The corresponding values in Degerman *et al*. (2007) were 38 and 50%. In addition, many European rivers are affected by water quality pressures. Our water quality pressure index (WQPI) shows a value of 59% for this pressure group. Degerman *et al*. (2007) reported similar results, as the loading of nutrients or organic matter was high in 49% of all sites.

We are aware of the restrictions of our approach to calculating pressures indices. A range of qualitative modelling techniques would be needed in further analyses, and a sensitivity analysis should be applied. Advanced techniques such as structural equation modelling might be considered in further research to evaluate linkages among pressure types, to link pressures to biota and, finally, to better cope with the complexity of running water ecosystems.

In terms of pressure combinations, Aarts *et al*. (2004) stated that water quality has improved markedly in European rivers. We also found that the more frequent impacts today are habitat loss and reduced hydrological connectivity.

### RTs

Across Europe, there is a large diversity in natural and human-induced characteristics of riverine systems. According to the IES (Solimini *et al*. 2006), the relationship between anthropogenic pressures and ecological status varies corresponding to the sensitivity of a river ecosystem to the related pressure combinations. Allan (2004) concluded that it is necessary to study pressure-impact relationships in geographically homogeneous areas to identify the relative influence of human and natural drivers on ecosystem responses. Using Illies ecoregions, we were able to detect various pressure combinations across different areas and RTs in Europe. Headwaters are less impacted than lowland rivers, and hydromorphological pressures are the most important for headwaters in the Alps, and multiple impacts are common in lowland rivers. The restoration of hydromorphological conditions is essential in headwaters and lowland rivers to meet the objectives of the WFD.

### GPI

The GPI was used to combine pressure intensity and multiple pressure effects for a large number of sampling sites across Europe. GPI can be a valuable tool because it classifies the pressure status of European rivers with a single standardised value. According to the IES (Solimini *et al*. 2006), such standardised tools are necessary to make profound political decisions and to successfully implement the WFD.

The European Committee for Standardization recently delivered a standard on hydromorphological pressure assessment (EN15843 2010; European Committee for Standardization 2010), but it covers only hydromorphological pressures and uses only verbal criteria descriptions. The GPI could be a more precise tool for WFD-related pressure classifications as a means to defining thresholds for human pressures similar to the classifications of ecological status.

We are aware that a reduction of dimensions during the compilation of the GPI leads to a loss of information and that the original dimensionality and the interaction between attributes has implications for actual interventions in the river systems. In addition, it has to be considered that our pressure data are based on different sources of information in various countries. However, even in its current version, the GPI clearly reflects the relationship between anthropogenic activities (human population density) and pressures affecting running water ecosystems. The GPI can be a helpful tool for river basin managers to identify impacted sites and to develop adequate restoration strategies for multiple impacted sites.

In comparison with North America, Paulsen *et al*. (2008) also stated that future assessments of US streams and rivers will have to include more comprehensive stressor (pressure) lists from each category. Although the most widespread stressors in the United States (nationally and in the three major regions of the eastern highlands, plains and lowlands, and the western area) are known to be N, *P*, riparian disturbance and streambed sediments, there is lack of common pressure information on hydrology, continuum disruption and combined pressure effects. In-depth analyses of relationships among various pressure types and linkages to biotic classifications may also yield a better understanding of restoration and mitigation requirements (Paulsen *et al*. 2008).

As Frissell & Ralph (1998) stated, maintaining the natural ecological values of river systems becomes increasingly difficult when the rate of change induced by human activities accelerates and the overall magnitude and persistence of the effects increases. According to Wohl *et al*. (2005), achieving restoration goals will be limited by a variety of scientific factors, such as unavailable information on critical ecosystem conditions or processes.

## Conclusions

1. Pressure-specific indices and the GPI enable standardised comparisons of the pressure status of ecoregions and RTs across Europe.
2. The degradation of European rivers is widespread. Single water quality pressures (W) are not common, but many sites are affected by hydromorphological pressures (HMC) or a combination of all pressures (W + HMC).
3. Hydromorphological pressures (HMC) are the key pressures in alpine regions and headwaters, whereas water quality pressures (W) and combined pressures (W + HMC) prevail in lowlands.
4. Although comprehensive pressure data in our study were available from national databases related to the WFD as well as other national and regional data sources, there are still data gaps for particular regions of Europe (e.g. southeastern countries) and in certain RTs (particularly in large rivers).
5. Our results constitute a baseline from which future trends can be evaluated. Especially in the context of river restoration, further work is needed to better identify the processes and effects that are relevant for the restoration of sites with multiple pressures and various pressure combinations.
